# The Effect of Tobacco on Youth

**Published:** 1883-10

**Authors:** 


### The Effect of Tobacco on Youth.

Dr. G. Decaisne has submitted to the Society of Public Medicine the results of some interesting observations concerning the effects due to the use of tobacco among boys. Thirty-eight youths were placed in his charge, whose ages varied from nine to fifteen, and who were in the habit of smoking, though the abuse of tobacco varied in each case. The effects of course also varied, but were very emphatic with twenty-seven out of the thirty-seven boys. With twenty-two patients there was a distinct disturbance of the circulation, bruit at the carotids, palpitation of the heart, deficiencies of digestion, sluggishness of the intellect, and a craving, more or less pronounced, for alcoholic stimulants. In thirteen instances there was an intermittent pulse. Analysis of the blood showed in eight cases a notable falling off in the normal number of red corpuscles. Twelve boys suffered frequently from bleeding of the nose. Ten complained of agitated sleep and constant nightmare. Four boys had ulcerated mouths, and one of the children became the victim of pulmonary phthisis, a fact which Dr. Decaisne attributed to the great deterioration of the blood produced by prolonged and excessive use of tobacco. As these children were all more or less lymphatic, it was not possible to establish a comparison according to temperament; but of course the younger the child the more marked were the symptoms, and the better-fed children were those that suffered least. Eight of the children in question were aged from nine to twelve years. Eleven had smoked for six months, eight for one year, and sixteen for more than two years. Out of eleven boys who were induced to cease smoking, six were completely restored to normal health after six months, while the others continued to suffer slightly for a year. Treatment with iron and quinine gave no satisfactory result, and it seems tolerably evident that the most effective, if not the only cure, is to at once forswear the habit, which to children in any case is undoubtedly pernicious.—*Lancet.* —*Scientific Am.*

